# Supplementary material for: End Sequence Analysis Toolkit (ESAT) expands the extractable information from single-cell RNA-seq data
Source: Genome Res. 2016 Oct;26(10):1397–410. doi: 10.1101/gr.207902.116 (PMC5052061; doi:10.1101/gr.207902.116)
Supplement: Supplemental Material [file supp_gr.207902.116_Supp_Materials.tar.gz › ESAT-esat_v0.1/doc/html/classbroad_1_1core_1_1datastructures_1_1_interval_tree_3_01_v_01_4-members.html]

ESAT: Member List


ESAT


- Main Page
- Related Pages
- Packages
- Classes
- Files

- Class List
- Class Index
- Class Hierarchy
- Class Members

All Classes Namespaces Files Functions Variables Enumerator Pages

- broad
- core
- datastructures
- IntervalTree< V >

broad.core.datastructures.IntervalTree< V > Member List

This is the complete list of members for broad.core.datastructures.IntervalTree< V >, including all inherited members.

|  |  |  |
| --- | --- | --- |
| clear() | broad.core.datastructures.IntervalTree< V > |  |
| doesOverlap(int start, int end) | broad.core.datastructures.IntervalTree< V > |  |
| find(Interval interval) | broad.core.datastructures.IntervalTree< V > |  |
| find(int start, int end) | broad.core.datastructures.IntervalTree< V > |  |
| findByIndex(int idx) | broad.core.datastructures.IntervalTree< V > |  |
| getIndex(Interval interval) | broad.core.datastructures.IntervalTree< V > |  |
| getIndex(int start, int end) | broad.core.datastructures.IntervalTree< V > |  |
| getSentinel() | broad.core.datastructures.IntervalTree< V > |  |
| isEmpty() | broad.core.datastructures.IntervalTree< V > |  |
| iterator() | broad.core.datastructures.IntervalTree< V > |  |
| iterator(Interval interval) | broad.core.datastructures.IntervalTree< V > |  |
| iterator(int start, int end) | broad.core.datastructures.IntervalTree< V > |  |
| max() | broad.core.datastructures.IntervalTree< V > |  |
| max(Interval interval) | broad.core.datastructures.IntervalTree< V > |  |
| max(int start, int end) | broad.core.datastructures.IntervalTree< V > |  |
| min() | broad.core.datastructures.IntervalTree< V > |  |
| min(Interval interval) | broad.core.datastructures.IntervalTree< V > |  |
| min(int start, int end) | broad.core.datastructures.IntervalTree< V > |  |
| minOverlapper(Interval interval) | broad.core.datastructures.IntervalTree< V > |  |
| minOverlapper(int start, int end) | broad.core.datastructures.IntervalTree< V > |  |
| numOverlappers(int start, int end) | broad.core.datastructures.IntervalTree< V > |  |
| overlappers(int start, int end) | broad.core.datastructures.IntervalTree< V > |  |
| overlappingValueIterator(int start, int end) | broad.core.datastructures.IntervalTree< V > |  |
| placeInTree(int start, int end, V value) | broad.core.datastructures.IntervalTree< V > |  |
| put(Interval interval, V value) | broad.core.datastructures.IntervalTree< V > |  |
| put(int start, int end, V value) | broad.core.datastructures.IntervalTree< V > |  |
| remove(Interval interval) | broad.core.datastructures.IntervalTree< V > |  |
| remove(int start, int end) | broad.core.datastructures.IntervalTree< V > |  |
| remove(int start, int end, V value) | broad.core.datastructures.IntervalTree< V > |  |
| reverseIterator() | broad.core.datastructures.IntervalTree< V > |  |
| reverseIterator(Interval interval) | broad.core.datastructures.IntervalTree< V > |  |
| reverseIterator(int start, int end) | broad.core.datastructures.IntervalTree< V > |  |
| setSentinel(V sentinel) | broad.core.datastructures.IntervalTree< V > |  |
| size() | broad.core.datastructures.IntervalTree< V > |  |
| toCollection() | broad.core.datastructures.IntervalTree< V > |  |
| toList() | broad.core.datastructures.IntervalTree< V > |  |
| valueIterator() | broad.core.datastructures.IntervalTree< V > |  |
| write(String fileName) | broad.core.datastructures.IntervalTree< V > |  |
| write(BufferedWriter bw) | broad.core.datastructures.IntervalTree< V > |  |


---

Generated on Fri Aug 15 2014 15:10:51 for ESAT by  

 1.8.7
